# Effect of steep-axis incisions on anterior and posterior corneal astigmatism and higher-order aberrations after cataract surgery

**DOI:** 10.3389/fmed.2026.1948208

**Published:** 2026-10-23

**Authors:** Kaiyue Du, Zhan Shen, Bowei Yuan, Yinan Liu, Xiaoyong Chen, Jing Hong

**Affiliations:** 1 Department of Ophthalmology, Peking University Third Hospital, Beijing, China; 2 Department of the Clinical Epidemiology Research Center, Peking University Third Hospital, Beijing, China

**Keywords:** cataract surgery, higher-order aberrations, phacoemulsification, steep-axis incision, surgically induced astigmatism

## Abstract

**Purpose:**

To compare the effects of steep-axis versus superior corneal incisions on visual acuity, astigmatism correction, and higher-order aberrations (HOAs) in patients undergoing phacoemulsification with intraocular lens implantation.

**Design:**

Prospective, case-control, single-center study.

**Methods:**

This study enrolled patients with preoperative corneal astigmatism <2.5 diopters. Outcomes included corrected distance visual acuity (CDVA), anterior and posterior corneal astigmatism, surgically induced astigmatism measured with Pentacam, and higher-order aberrations (HOAs) assessed using Pentacam and iTrace at 1 and 3 months postoperatively.

**Results:**

A total of 148 right eyes from 148 patients were analyzed, including 56 in the steep-axis incision group and 92 in the superior-incision group. CDVA did not differ significantly between groups at 3 months (*p* = 0.617). Steep-axis incisions produced significantly lower anterior corneal astigmatism (*p* = 0.006) and a greater flattening effect (*p* < 0.001) at 3 months. No significant intergroup differences were observed in total HOAs of the total cornea, anterior cornea, or posterior cornea within the 6-mm zone by either Pentacam or iTrace. Subgroup analysis of steep-axis orientations (with-the-rule, against-the-rule, oblique) revealed no significant differences in outcomes.

**Conclusion:**

Steep-axis incisions effectively reduce anterior corneal astigmatism without increasing HOAs, representing a practical option for correcting mild-to-moderate astigmatism in cataract surgery.

## Introduction

Cataracts are the leading cause of reversible blindness worldwide, which has made cataract surgery the most common surgery in the world. Since the introduction of phacoemulsification in 1967, ongoing technological advancements have transformed cataract surgery into a refractive procedure (1). The objective of refractive cataract surgery extends beyond merely restoring visual acuity to pursuing the enhancement of visual quality.

The postoperative visual quality depends on several optical factors, the most important of which are lower-order aberrations. Among them, postoperative astigmatism affects the visual quality and efficacy of refractive cataract surgery, which is caused by a combination of preoperative corneal astigmatism and surgical-induced astigmatism (SIA) resulting from the surgical incision (2, 3). SIA refers to corneal astigmatism induced by the incision made during cataract surgery. SIA is associated with factors such as the incision's length, location, configuration, and tunnel length (4, 5). The steep-axis corneal incision is performed on the steepest corneal meridian. The resulting SIA provides a corrective effect for pre-existing corneal astigmatism. A meta-analysis has indicated that treatment of astigmatism can be achieved by both toric intraocular lenses (IOLs) and steep-axis corneal incisions (6). There was no evidence of an important difference in postoperative visual acuity or quality of life between the techniques. But the steep-axis corneal incision is currently controversial. Some scholars suggested that steep-axis incisions flatten steep meridians while steepening flat meridians, making it difficult to accurately predict postoperative astigmatism or other low-order aberrations (7).

In addition, higher-order aberrations (HOAs) are also important for visual quality (8, 9). Previous studies have observed significantly increased corneal HOAs after cataract surgery (9–11). For example, previous studies have indicated that superior incisions can induce vertical or horizontal coma, while temporal incisions exert minimal influence. Therefore, further exploration is needed to determine whether the use of steep axis incisions will induce new higher-order aberrations that may affect the postoperative visual quality of patients. Therefore, the corrective effect of steep-axis incisions on postoperative astigmatism requires further evaluation.

This study aims to explore the correction effect of steep axis incisions on astigmatism compared to traditional incisions, as well as the potential introduction of additional low or high-order aberrations.

## Methods

### Participants

This prospective, real-world study included patients with age-related cataract undergoing phacoemulsification and IOL implantation between July 2024 and December 2024. All participants provided informed consent. The study protocol was approved by the Medical Science Research Ethics Committee of Peking University Third Hospital (Protocol No. M2022067) and adhered strictly to the principles of the Declaration of Helsinki. All participants provided written informed consent.

Patients were included if they had preoperative corneal astigmatism of less than 2.5 diopters (D). Surgical planning was determined according to the measured preoperative corneal astigmatism under real-world clinical conditions. A superior incision was applied for corneal astigmatism <0.5 D; a steep-axis incision was selected for 0.5–1.0 D; and for 1.0–2.5 D, a toric IOL was implanted in combination with either a steep-axis or a superior incision. Exclusion criteria encompassed the presence of ocular surface disorders (such as severe dry eye, ectropion, entropion, or trichiasis), corneal disease, uveitis, glaucoma, retinopathy, neurological disorders, a history of ocular trauma or surgery, systemic diseases (including diabetes mellitus, thyroid disease, or autoimmune disorders), significant intraoperative or postoperative complications, and incomplete follow-up data. Only the right eye of the participants was used in the study.

### Clinical data measurement

All patients underwent a comprehensive ophthalmologic evaluation within two weeks before surgery. Preoperative assessments included uncorrected distance visual acuity, corrected distance visual acuity (CDVA), manifest and cycloplegic refraction, slit-lamp biomicroscopy, intraocular pressure measurement, endothelial cell density, ultrasound A- and B-scan, dilated indirect fundoscopy, anterior segment optical coherence tomography (Visante; Zeiss, Oberkochen, Germany), and ocular biometry using the IOL Master 700 (Zeiss, Jena, Germany). Additional examinations comprised corneal tomography (Pentacam; Oculus, Washington, DC, USA) and ray tracing wavefront aberrometry (iTrace; Tracey Technologies, Houston, TX, USA).

Preoperative markings for the tunnel and sideport incisions were made at the slit lamp while the patient was seated to minimize cyclotorsion in the supine position. SIA was calculated using vector analysis according to the Alpins method (12). SIA magnitude represented the magnitude of the induced astigmatic vector, while its components included the flattening effect (astigmatism reduction along the preoperative axis), torque (the rotational component of induced astigmatic change), and centroid SIA (the mean induced astigmatism vector). Absolute torque represented the magnitude of torque irrespective of its direction.

Postoperative follow-up was performed at 1 week, 1 month, and 3 months. At each visit, visual acuity, manifest refraction, slit-lamp biomicroscopy, and intraocular pressure were assessed. Pentacam imaging and iTrace aberrometry were performed at 1 and 3 months postoperatively.

#### Surgical procedures

Intraoperative biometry data from the IOLMaster 700 were integrated and aligned with the Callisto Eye surgical navigation system, which displayed incision axis markers to guide the intended incision location. A 3.0 mm triplanar clear corneal main incision and a 0.6 mm side-port incision were created. An ophthalmic viscosurgical device (OVD) was injected into the anterior chamber, and continuous curvilinear capsulorhexis was performed under navigation guidance, using a corneal plane diameter of 5.5–6.0 mm as the reference. Hydrodissection and hydrodelineation were subsequently performed. Cataract removal was achieved via phacoemulsification using the Centurion Vision System (Alcon Laboratories, Fort Worth, TX, USA). After reinjecting OVD into the anterior chamber, an IOL was implanted into the capsular bag and centered with the aid of the navigation system. Then, residual OVD was removed, and the incisions were sealed by stromal hydration. At the conclusion of surgery, tobramycin–dexamethasone ointment was applied, and the operated eye was dressed with an eye pad.

### Statistical analysis

Statistical analyses were conducted using R software (version 4.4.3; R Foundation for Statistical Computing, Vienna, Austria). Categorical variables were reported as counts, and continuous variables as means ± standard deviations. The normality of continuous variables was assessed using the Shapiro–Wilk test. For normally distributed data, the independent Student's *t*-test was applied; for non-normally distributed data, the exact Wilcoxon rank-sum test was used. For subgroup analyses, because at least one group for each variable violated the assumption of normality, the non-parametric Kruskal–Wallis test was employed for intergroup comparisons. Categorical data were compared using the Chi-square test with Bonferroni adjustment. A *p* < 0.05 was considered statistically significant.

## Results

A total of 148 eyes from 148 patients underwent cataract surgery during the study period. Complete data were available for 56 eyes in the steep-axis incision group and 92 eyes in the superior-incision group. The clinical characteristics of both groups are presented in Table 1. Baseline clinical data were comparable between the two groups. No patient required Nd: YAG capsulotomy during the study period.

**Table 1:** Baseline clinical characteristics of the two groups.

	Steep-axis incision	Superior incision	*P* value
No. eyes	56	92	
Sex, M, %(n)	23.21 (13)	38.04 (35)	0.091^a^
Age, years	68.6 ± 8.6	69.9 ± 10.1	0.118^b^
CDVA, logMAR	0.38 ± 0.25	0.38 ± 0.23	0.672^b^
Astigmatism, D	0.80 ± 0.55	0.89 ± 0.42	0.060^b^
Astigmatism meridian,°	102.86 ± 47.50	92.45 ± 65.38	0.350^b^
Mean keratometry, D	44.13 ± 1.91	44.14 ± 2.12	0.525^b^
Mean keratometric astigmatism, D	0.81 ± 0.54	0.81 ± 0.42	0.581^b^
Central corneal thickness, μm	544.3 ± 30.1	545.2 ± 32.6	0.706^b^
Pupil diameter, mm	5.34 ± 0.80	5.26 ± 0.93	0.599^c^
Anterior chamber depth, mm	2.99 ± 0.40	3.04 ± 0.50	0.495^c^
White to white, mm	11.23 ± 0.39	11.21 ± 0.44	0.817^c^

Continuous variables are presented as means ± standard deviations, and categorical variables as percentages (n).

a Result from the Chi-square test with Bonferroni adjustment.

b Results from exact Wilcoxon rank-sum test.

c Results from the independent Student's t-test.

M, male; L,  left; CDVA, corrected distance visual acuity; logMAR, logarithm of the minimum angle of resolution; D, diopter.

### Visual acuity

A significant improvement in CDVA was observed in both groups at 3 months postoperatively (*p* < 0.001 for both). No significant differences in CDVA were detected between the two groups preoperatively (*p* = 0.672) or at the 1-week, 1-month, and 3-month follow-up visits (*p* = 0.803, 0.878, and 0.617, respectively) (Figure 1).

**Figure 1:**
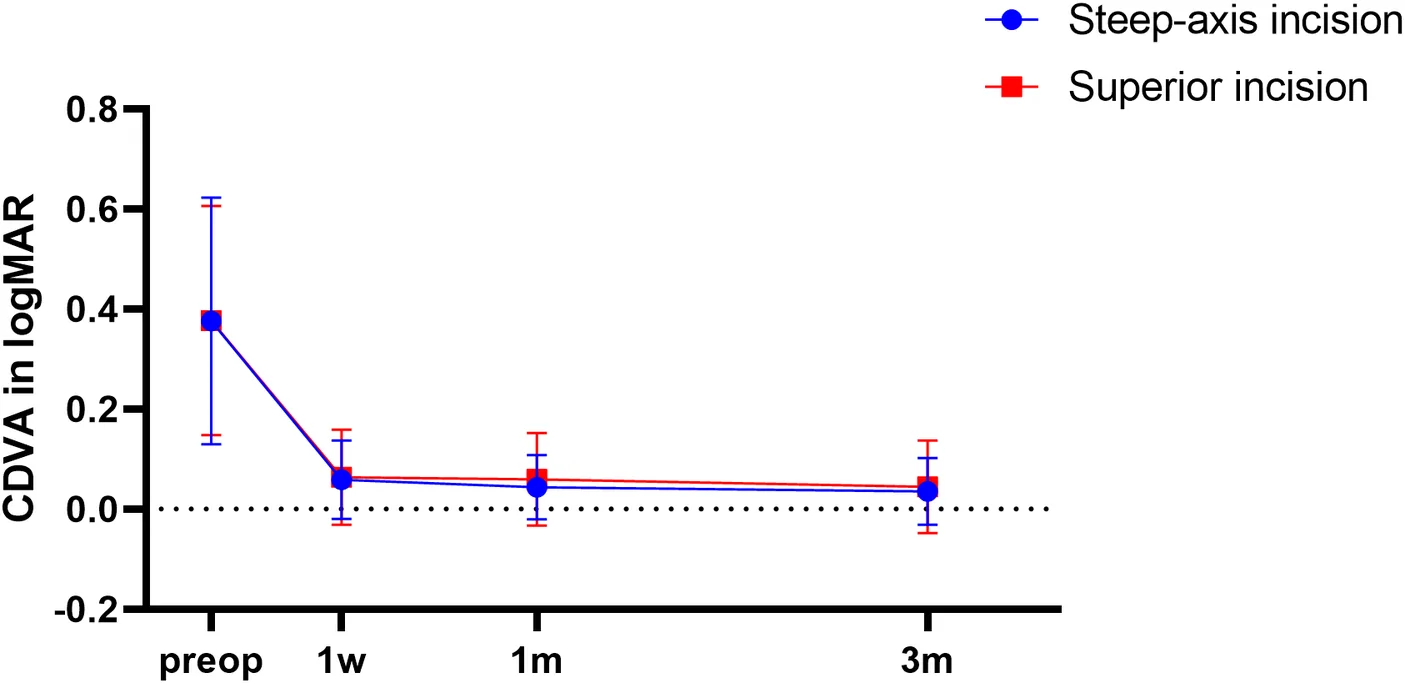
CDVA at different time points in the two groups. Statistical comparisons were performed using the exact Wilcoxon rank-sum test. CDVA = corrected distance visual acuity; logMAR = logarithm of the minimum angle of resolution; preop = preoperative.

### Corneal astigmatism

No significant differences in anterior corneal astigmatism were observed between the two groups preoperatively, nor within each group between pre- and postoperative measurements. However, anterior corneal astigmatism was significantly lower in the steep-axis incision group compared with the superior incision group at the 1-month and 3-month follow-up visits (*p* = 0.030 and 0.006, respectively), with between-group differences of 0.34 D and 0.27 D, respectively. For posterior corneal astigmatism, no significant differences were detected within groups (pre- vs. postoperative) or between groups at any time point (Figure 2).

**Figure 2:**
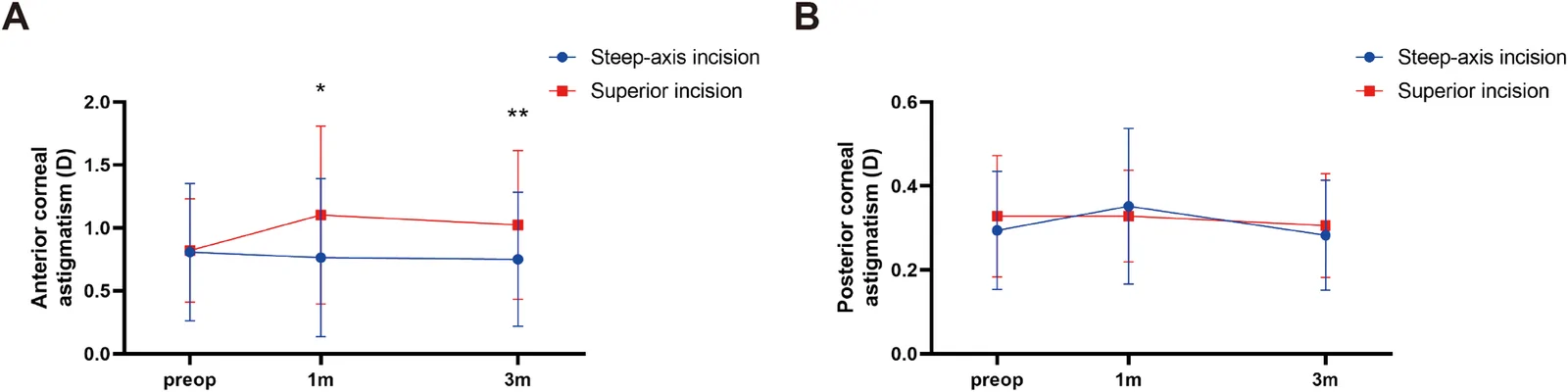
Corneal astigmatism at different time points in the two groups. **(A)** anterior corneal astigmatism. **(B)** posterior corneal astigmatism. Statistical comparisons were performed using the exact Wilcoxon rank-sum test. preop = preoperative; D = diopter. *p < 0.05, **p < 0.01.

### Corneal SIA

At 1 and 3 months postoperatively, the flattening effect of both the anterior and posterior cornea differed significantly between the two groups (*p* = 0.040, 0.025, <0.001, and 0.042, respectively). No other SIA–related parameters on the anterior or posterior corneal surfaces showed significant differences between the groups (Table 2).

**Table 2:** SIA assessment results.

	Steep-axis incision	Superior incision	*P* value
Corneal SIA assessments (postop 1 m)			
Anterior cornea			
SIA magnitude, D	0.77 ± 0.47	0.88 ± 0.51	0.335^a^
Centroid SIA, magnitude @ axis	0.31 @ 109.0	0.71 @ 87.6	NA
Flattening effect, D	0.42 ± 0.66	0.07 ± 0.81	0.040^b^
Torque, D	0.03 ± 0.46	0.03 ± 0.61	0.355^a^
Absolute torque, D	0.36 ± 0.28	0.47 ± 0.39	0.298^a^
Posterior cornea			
SIA magnitude, D	0.14 ± 0.12	0.11 ± 0.07	0.459^a^
Centroid SIA, magnitude @ axis	0.05 @ 3.0	0.03 @ 126.5	NA
Flattening effect, D	−0.04 ± 0.14	0.02 ± 0.10	0.025^a^
Torque, D	−0.01 ± 0.12	0.03 ± 0.08	0.231^a^
Absolute torque, D	0.07 ± 0.09	0.06 ± 0.06	0.839^a^
Corneal SIA assessments (postop 3 m)			
Anterior cornea			
SIA magnitude, D	0.56 ± 0.33	0.65 ± 0.32	0.104*^a^*
Centroid SIA, magnitude @ axis	0.18 @ 124.8	0.42 @ 90.1	NA
Flattening effect, D	0.28 ± 0.43	−0.01 ± 0.56	< 0.001*^b^*
Torque, D	0.07 ± 0.40	−0.00 ± 0.46	0.341*^b^*
Absolute torque, D	0.31 ± 0.26	0.36 ± 0.28	0.387*^a^*
Posterior cornea			
SIA magnitude, D	0.11 ± 0.07	0.12 ± 0.06	0.619*^a^*
Centroid SIA, magnitude @ axis	0.03 @ 128.1	0.06 @ 98.8	NA
Flattening effect, D	0.02 ± 0.10	0.05 ± 0.09	0.042*^a^*
Torque, D	0.02 ± 0.07	0.01 ± 0.08	0.261*^b^*
Absolute torque, D	0.06 ± 0.05	0.07 ± 0.05	0.300*^a^*

a Results from the exact Wilcoxon rank-sum test.

b Results from the independent Student's t-test.

NA, not applicable; postop, postoperative; SIA, surgically induced astigmatism; D, diopter.

### Corneal HOAs

No significant differences in total, anterior, or posterior corneal HOAs were observed between the two groups at the preoperative visit or at 1 and 3 months postoperatively (Figure 3). At 1 month postoperatively, the 6 mm total corneal HOAs measured by Pentacam significantly increased compared with preoperative values in both the steep-axis incision group (*p* = 0.005) and the superior incision group (*p* = 0.028). In the steep-axis incision group, significant increases were also detected at 1 month in 6 mm anterior and posterior corneal HOAs measured by Pentacam (*p* = 0.003 and 0.040, respectively), as well as in 3 mm and 4 mm total corneal HOAs measured by iTrace (*p* = 0.014 and 0.009, respectively) (Figure 4). By 3 months postoperatively, none of the HOA parameters differed significantly from preoperative values in either group.

**Figure 3:**
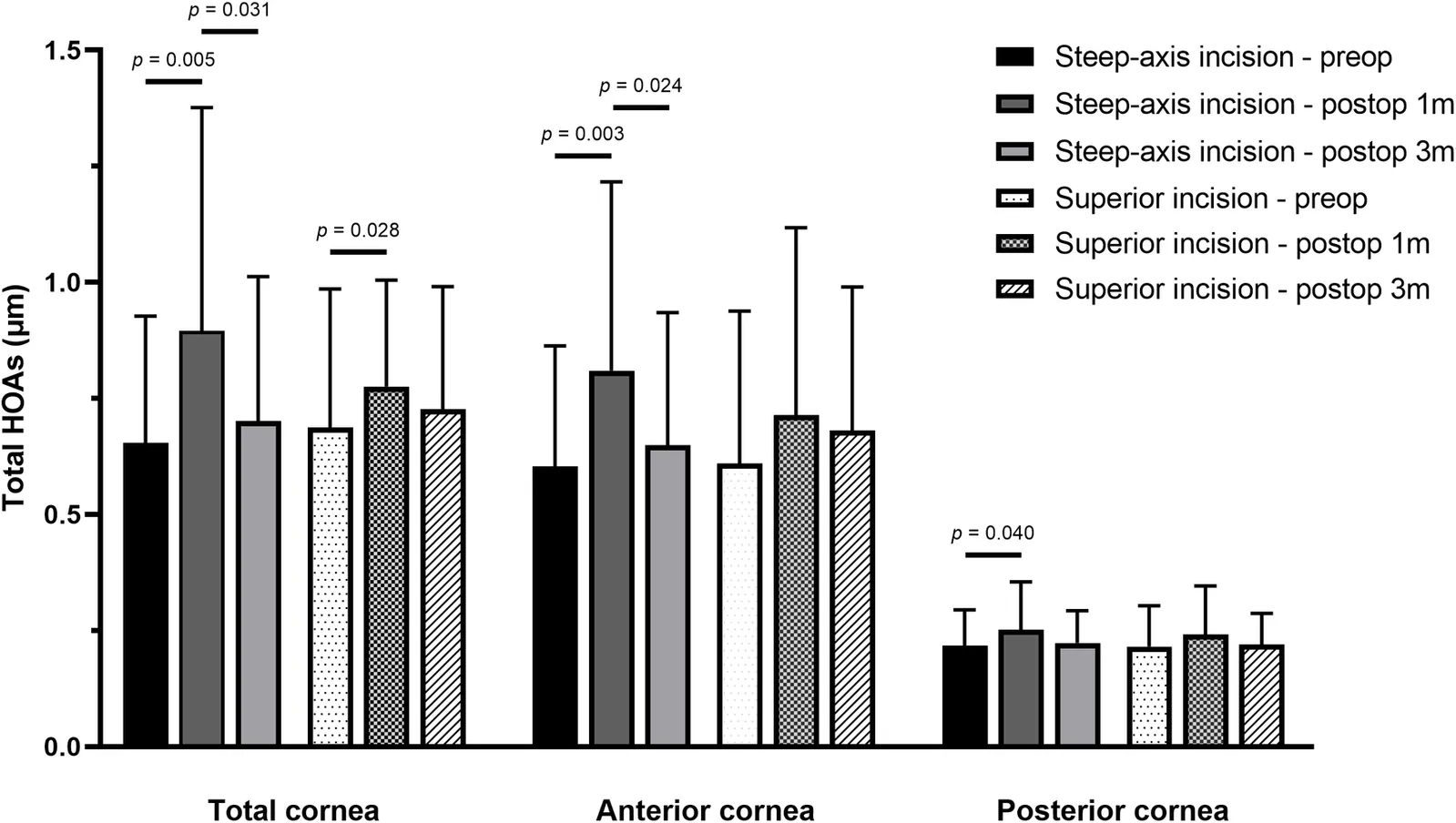
Total HOAs of the total cornea, anterior cornea, and posterior cornea measured by pentacam over the 6 mm analytical zone, presented by group. Statistical analysis was performed using the exact Wilcoxon rank-sum test. preop = preoperative; postop = postoperative; HOAs = higher-order aberrations.

**Figure 4:**
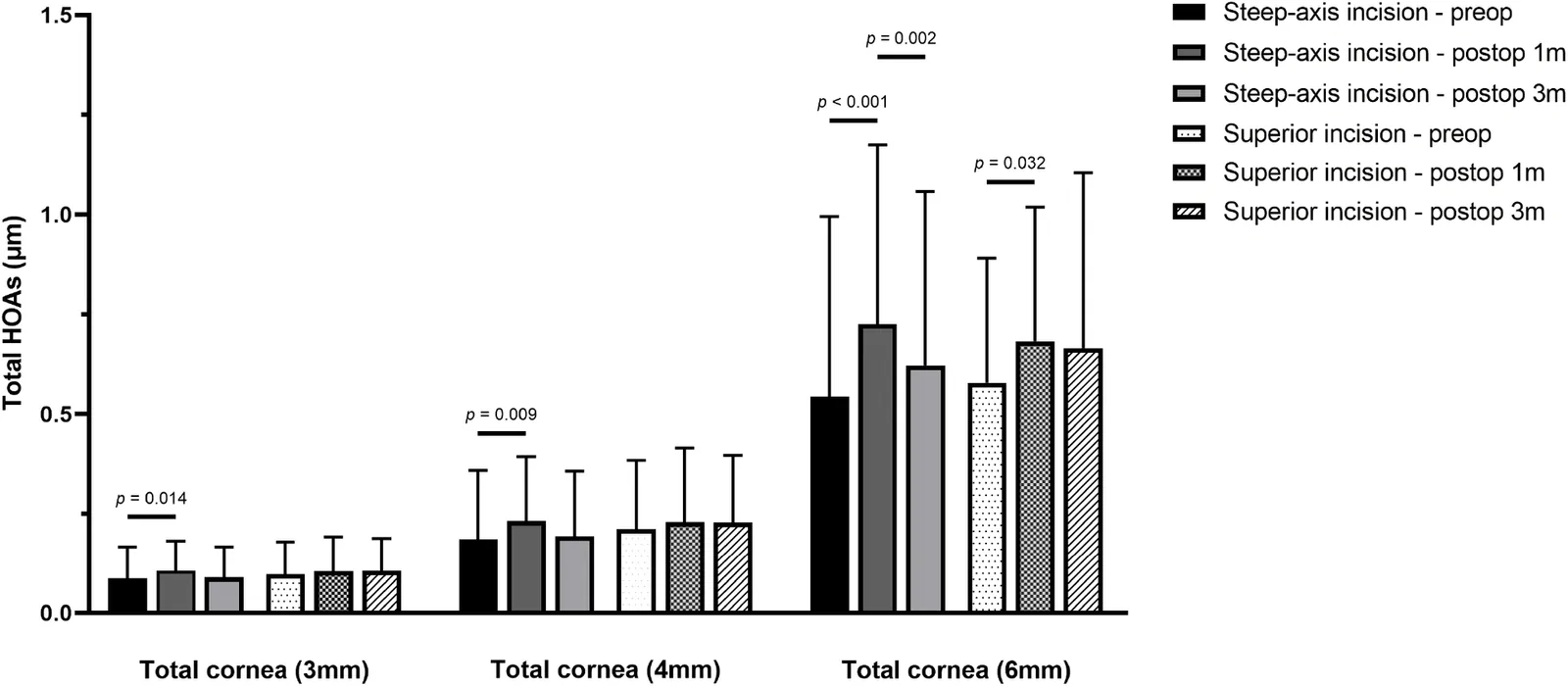
Total HOAs measured by iTrace over the 3 mm, 4 mm, and 6 mm analytical zones, presented by group. Statistical analysis was performed using the exact Wilcoxon rank-sum test. preop = preoperative; postop = postoperative; HOAs = higher-order aberrations.

### Subgroup analyses in the steep-axis incision group

Patients in the steep-axis incision group were classified according to the axis of the steepest corneal meridian as with-the-rule (WTR; 60°–120°), against-the-rule (ATR; 0°–30° or 150°–180°), or oblique (>30° to <60° or >120° to <150°) (13) At 3 months postoperatively, no significant differences were found among the three subgroups in any primary outcome, including CDVA, anterior and posterior corneal astigmatism, SIA magnitude, or total corneal HOAs over the 6-mm zone measured by either Pentacam or iTrace (Table 3).

**Table 3:** Main outcomes at 3 months after surgery in the steep-axis incision group, stratified by incision orientation.

Parameter	With-the-rule	Against-the-rule	Oblique	*P* value	Overall effect size (95% CI)
n	33	13	10		
CDVA, logMAR	0.04 ± 0.07	0.03 ± 0.09	0.02 ± 0.03	0.236	0.031 (0–0.104)
Anterior corneal astigmatism, D	0.82 ± 0.54	0.55 ± 0.39	0.69 ± 0.60	0.288	0.043 (0–0.137)
Posterior corneal astigmatism, D	0.30 ± 0.14	0.28 ± 0.13	0.24 ± 0.10	0.398	0.028 (0–0.109)
SIA magnitude, anterior cornea, D	0.62 ± 0.30	0.53 ± 0.42	0.43 ± 0.29	0.166	0.072 (0–0.171)
SIA magnitude, posterior cornea, D	0.11 ± 0.07	0.09 ± 0.06	0.12 ± 0.05	0.387	0.030 (0–0.112)
Total cornea HOAs 6 mm, Pentacam, μm	0.72 ± 0.33	0.68 ± 0.32	0.68 ± 0.27	0.802	0.012 (0–0.082)
Total cornea HOAs 6 mm, iTrace, μm	0.60 ± 0.18	0.57 ± 0.15	0.59 ± 0.17	0.509	0.021 (0–0.094)

CDVA, corrected distance visual acuity; logMAR, logarithm of the minimum angle of resolution; D, diopter; SIA, surgically induced astigmatism; HOAs, higher-order aberrations.

## Discussion

This study compared the visual and refractive outcomes of steep-axis versus non-steep-axis superior corneal incisions during phacoemulsification with IOL implantation in patients with age-related cataract. Surgical planning and patient allocation were determined according to preoperative corneal astigmatism, and baseline corneal astigmatism was comparable between the two groups. As a real-world investigation, the study followed routine practice, with patients with lower astigmatism typically receiving monofocal IOLs and those with higher astigmatism receiving toric IOLs. IOL selection was considered an internal optical factor and is unlikely to have significantly influenced subsequent analyses of corneal astigmatism or HOAs, as prior studies consistently show that corneal incisions have a far greater impact on corneal morphology than IOL type (14). Thus, the grouping strategy in this study reflects standard clinical decision-making and supports the generalizability of our findings to broader patient populations and real-world treatment settings.

Both incision groups produced significant improvements in CDVA by 3 months postoperatively, with no significant intergroup differences at any time point. Notably, anterior corneal astigmatism was significantly lower in the steep-axis incision group than in the superior incision group at both 1 and 3 months postoperatively, whereas posterior corneal astigmatism remained unchanged. These findings support the notion that on-axis incisions can effectively reduce preexisting corneal astigmatism (15). However, the between-group differences were relatively small (0.34 D at 1 month and 0.27 D at 3 months), and their clinical relevance should therefore be interpreted cautiously.

Regarding SIA, prior studies have reported that superior incisions generate the greatest SIA, nasal incisions produce intermediate levels, and temporal incisions produce the least (16, 17). Sonmez and Karaca also indicated that superior incisions with longer tunnels had a higher astigmatic effect when compared to temporal incisions with long tunnels or temporal and superior incisions with short tunnels (4).

Consistent with these reports, we observed that superior incisions induced slightly more SIA than steep-axis incisions at both 1 and 3 months, although the differences were not statistically significant. Another study suggested that steep-axis incisions flatten the steep meridian while steepening the flat meridian, and in eyes with less than 2.6 D of preoperative astigmatism, on-axis incisions produced only slightly greater flattening than temporal incisions, albeit with significantly higher absolute torque (7).

In contrast, our findings indicated that steep-axis incisions achieved a more favorable flattening effect on the anterior cornea compared with superior incisions, without significant differences in absolute torque. Collectively, these results suggest that steep-axis incisions may offer a clinical advantage in reducing residual anterior corneal astigmatism without introducing additional rotational instability.

Beyond lower-order aberrations, HOAs also play an important role in postoperative optical quality (18). Previous studies have shown that superior incisions can induce vertical or horizontal coma depending on the orientation of the steep meridian, potentially compromising optical quality, whereas temporal incisions may minimize corneal HOAs (19). Another study reported that superior incisions exert the greatest effect on HOAs, particularly vertical coma and trefoil, followed by nasal incisions, with temporal incisions exerting minimal influence; however, the effect of oblique incisions remains uninvestigated (20). The greater impact of superior incisions has been attributed to anatomical and biomechanical factors, including the smaller vertical corneal diameter, the proximity of the superior limbus to the visual axis, and pressure from the upper eyelid (20). Other reports suggest that non-steep-axis incisions may increase irregular astigmatism, reducing visual quality, whereas steep-axis incisions are associated with more favorable HOA outcomes, though the precise locations of non–steep-axis incisions were not specified (21).

In our study, HOAs increased transiently at 1 month postoperatively in both groups but returned to preoperative levels by 3 months, likely reflecting early corneal edema at the central cornea and incision site (22). Importantly, no significant intergroup differences were observed, indicating that incision location did not substantially affect long-term optical quality beyond its impact on anterior corneal astigmatism. Compared with superior incisions, steep-axis incisions primarily influenced astigmatism, while their effect on HOAs was less pronounced and warrants further investigation.

Subgroup analysis of steep-axis incisions according to meridian orientation (with-the-rule, against-the-rule, and oblique) revealed no significant differences in visual acuity, astigmatism correction, or HOAs at 3 months, suggesting that the beneficial effect of steep-axis incision placement was consistent across different astigmatism axes. However, interpretation is limited by the imbalance in subgroup sizes, particularly the smaller numbers in the against-the-rule and oblique groups, which may have reduced statistical power. Future studies should include more patients in these subgroups to confirm these findings.

Several limitations should be acknowledged. First, this was a non-randomized observational study in which treatment allocation was guided by preoperative corneal astigmatism and clinical considerations, which may have introduced selection bias and residual confounding. Second, the unequal subgroup sizes limited the statistical power to detect between-group differences, and the nonsignificant findings should not be interpreted as evidence of equivalence. Third, the groups were unequal in size, and the follow-up period was relatively short. Direct comparisons with temporal or nasal non–steep-axis incisions were also not performed because incision selection was based on clinical considerations and surgeon preference. Therefore, the findings should be interpreted with caution. Future prospective studies with larger, balanced cohorts, longer follow-up, and standardized or randomized allocation, with appropriate adjustment for baseline astigmatism, are needed to validate these findings and further evaluate the potential benefits of combining steep-axis incisions with toric IOL implantation in eyes with higher preoperative astigmatism.

Overall, our findings indicate that steep-axis incisions provide an advantage in controlling anterior corneal astigmatism without compromising visual acuity or optical quality, supporting their use as a reasonable surgical option for patients with mild to moderate preoperative astigmatism.

## Data Availability

The original contributions presented in the study are included in the article/Supplementary Material, further inquiries can be directed to the corresponding author.
